# A European multi-language initiative to make the general population aware of independent clinical research: the European Communication on Research Awareness Need project

**DOI:** 10.1186/s13063-015-1146-7

**Published:** 2016-01-12

**Authors:** Paola Mosconi, Gerd Antes, Giorgio Barbareschi, Amanda Burls, Jacques Demotes-Mainard, Iain Chalmers, Cinzia Colombo, Silvio Garattini, Christian Gluud, Gill Gyte, Catherine Mcllwain, Matt Penfold, Nils Post, Roberto Satolli, Maria Rosa Valetto, Brian West, Stephanie Wolff

**Affiliations:** Laboratory for Medical Research and Consumers involvement, Department of Public Health, IRCCS Istituto di Ricerche Farmacologiche Mario Negri, Via Giuseppe La Masa 19, 20156 Milano, Italy; Medical Centre – University of Freiburg, Universitaetsklinikum Freiburg, German Cochrane Centre, Berliner Allee 29, 79110 Freiburg, Germany; European AIDS Treatment Group, Place Raymond Blyckaerts, 13, 1050 Brussels, Belgium; Oxford Radcliffe Hospitals NHS Trust, Headley Way, Headington, Oxford, OX3 9DU UK; INSERM-Institute National de la Santé et de la Recherche Médicale, 101 Rue de Tolbiac, Paris, 75654 France; The Copenhagen Trial Unit, Centre for Clinical Intervention Research, Rigshospitalet, Copenhagen University Hospital, 2100 Copenhagen, Denmark; Cochrane Consumer Network, UK, The Cochrane Collaboration LBG, Summertown Pavilion 18-24 Middle Way, Oxford, OX2 7LG UK; KKS-Network, c/o University Hospital of Cologne, Kerpener Strasse 62, Cologne, Germany; Zadig, Science and Health communication, Via Ampère 59, 20131 Milano, Italy

**Keywords:** Independent clinical trial, Patients, Lay people, Health research, Information, Access to information

## Abstract

**Background:**

The ECRAN (European Communication on Research Awareness Needs) project was initiated in 2012, with support from the European Commission, to improve public knowledge about the importance of independent, multinational, clinical trials in Europe.

**Methods:**

Participants in the ECRAN consortium included clinicians and methodologists directly involved in clinical trials; researchers working in partnership with the public and patients; representatives of patients; and experts in science communication. We searched for, and evaluated, relevant existing materials and developed additional materials and tools, making them freely available under a Creative Commons licence.

**Results:**

The principal communication materials developed were:

1. A website (http://ecranproject.eu) in six languages, including a Media centre section to help journalists to disseminate information about the ECRAN project
2. An animated film about clinical trials, dubbed in the 23 official languages of the European Community, and an interactive tutorial
3. An inventory of resources, available in 23 languages, searchable by topic, author, and media type
4. Two educational games for young people, developed in six languages
5. Testing Treatments interactive in a dozen languages, including five official European Community languages
6. An interactive tutorial slide presentation testing viewers’ knowledge about clinical trials

**Conclusions:**

Over a 2-year project, our multidisciplinary and multinational consortium was able to produce, and make freely available in many languages, new materials to promote public knowledge about the importance of independent and international clinical trials. Sustained funding for the ECRAN information platform could help to promote successful recruitment to independent clinical trials supported through the European Clinical Research Infrastructure Network.

## Background

At the end of 2013, the European Clinical Research Infrastructure Network (ECRIN) was awarded the status of European Research Infrastructure Consortium (ERIC) to continue designing and conducting independent and multinational clinical trials throughout Europe [1]. In 2014, the European Parliament approved the new Clinical Trials Regulations intended to make it easier to conduct multinational clinical trials [2, 3]. However, there has not been yet any strategic coordination for communicating the clinical and societal benefits of clinical trials to patients and the European public.

The public and patients have been reached with different messages and materials used by many communicators: research centres; patients’ advocacy groups; and pharmaceutical companies. The quality of the existing disparate communication activities ranges from reliable information to deliberate misinformation, and efforts to engage the public in understanding health research at the European level are rare. For example, the website of the European Science Foundation provides no materials addressed to the public, and there is no common strategy on how to communicate the importance of independent randomised clinical trials. Although randomised clinical trials and systematic reviews of such trials are recognised as essential for providing reliable evidence to inform medical practice, patients and the public more generally, still lack knowledge and awareness of the importance of these research methods [4–7].

In most European countries clinical trials are rarely the focus of public debate or patient organisations [8]. Even more rarely are research priorities debated by researchers, clinicians, and patients [9]. The involvement of the European public in supporting independent clinical trials to inform healthcare decisions varies among countries, misconceptions about clinical trials are frequent [10, 11], and the level of participation in clinical trials is low [4].

Increasing the awareness of *independent* clinical trials among patients and the public, supporting patient-oriented trials and avoiding wasteful research are important goals to promote better research for better healthcare [12–15]. In Europe, for example, Germany’s drug regulators suspended the use of 29 generic drugs on suspicion that they were given marketing licences based on forged medical reports of human trials supplied by GVK Biosciences, an Indian contract research organisation, and authorities in France and Belgium have taken similar action. The European Medicines Agency is reported to believe that GVK Biosciences has been systematically manipulating its medical studies for a number of years. The suspension led to a major outcry by pharmacists who were faced with disgruntled clients who still wanted to buy their drugs. These events illustrated why the public and patients need to understand the importance of *independent* clinical trials, as pharmacists complained that they found it impossible to explain the reasons to customers, in particular because the authorities had made clear that there was no threat to patients’ health [16].

Although ECRIN exists to design, coordinate, and facilitate the work of European clinical trialists, it lacks information materials for patients and the general population. In 2012, to address this gap and support the participation of patients in independent and multinational clinical trials in Europe, the European Commission funded the European Communication on Research Awareness Needs (ECRAN) project. ECRAN’s principal aims are to inform the public and patients about (1) the importance of independent clinical trials driven by healthcare needs; (2) the need for transparency and optimal use of clinical trial data; and (3) multinational cooperation, taking advantage of Europe’s population size and diversity. This article summarises the different phases of the ECRAN project and presents the materials developed.

## Methods

**Fig. 1:**
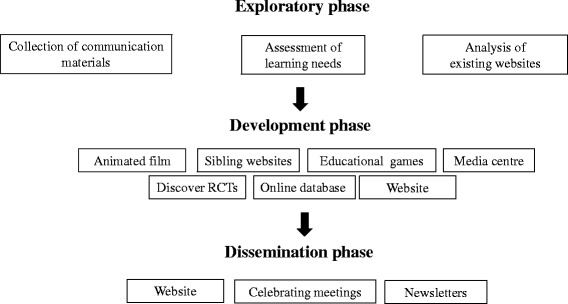
European Communication on Research Awareness Needs (ECRAN) project overview. Legend: *RCT* randomised clinical trials

The project started on 1 September 2012 and ended on 31 August 2014; as reported in Fig. 1 three work phases characterised the project: an exploratory phase aimed to collect opinion from consumers and materials already available; a development phase aimed to produce new ECRAN materials; and the dissemination phase aimed to publicise the results.

### Consortium

A multidisciplinary consortium handled the project: clinicians and methodologists involved in clinical trials; researchers working in partnership with the public and patients; representatives of patients; and experts in science communication. The materials developed took advantage of this diversity. The documents were reviewed to develop simple, readable materials for the public, and to be potentially useful for clinicians and researchers. For some materials, such as the website and the educational games, there was preliminary user-testing.

The consortium agreed to promote the following messages:

- The importance of public understanding of the need for, and basic principles of, clinical trials, fostering active involvement of the public, patients, and their representatives in trial design
- The need for independent clinical trials driven by healthcare and patients’ needs, not only on drugs but on all interventions, to optimise strategies, support evidence-based clinical practices, and reduce healthcare inequalities
- The need for transparency and optimal use of clinical trial data to allow their use in re-analysis, new analysis, and meta-analysis, but also to prevent bias in presenting and analysing results
- The need for multinational cooperation, taking advantage of Europe’s population size, diversity, and medical expertise

### Exploratory phase: survey and database

Public users of health information in the European Union were surveyed to see what they already understood about clinical trials, and what they wanted to learn more about. The survey also aimed to identify differences between the perspectives and approaches of the public and those of experts concerning what they need to learn about clinical trials, and to systematically identify open access resources that explain important aspects of clinical trials. The purpose was to inform subsequent resources development and enable new resources to be tailored to the public’s self-identified needs.

For the survey the ECRAN experts developed a list of the important concepts needed to understand clinical trials. The survey was piloted informally with a few people from the Cochrane Consumer Network, without further validation, because these concepts are well-established. The questions used were multiple-choice, free-text and matrix-type.

The surveys were distributed through: (a) PatientView, an organisation that holds a database of patient groups, through emails to 20,000 English-speaking patient groups across Europe, and (b) National Health Service Choices, a website funded by the English Department of Health, dedicated to informing the public on health issues. No reminders or follow-up emails were sent to non-responders.

Open access resources that explained important aspects of clinical trials were sought through (1) professional contacts (e.g. survey of members of the Network to Support Understanding of Health Research); (2) online searches by ECRAN partner organisations; (3) clinical trials search engines or trial registers; and (4) websites communicating information about clinical trials to patients, the public and/or professionals, developed by governments, institutions, and patient organisations. Resources focusing on one or more of ECRAN’s key messages were collated. These resources were catalogued in an online Koha database, so further data could be collected and the resources reviewed and evaluated.

Koha was chosen as it is a highly developed and reliable open source library cataloguing system that has templates for the major categories of resources (e.g. videos), the ability to add or edit templates, a flexible front-end design, Web 2.0 functionality for rating resources, and the ability to use iFrames to host particular content elsewhere to facilitate dissemination. The Koha database is also searchable in all 23 European languages. It works through a search engine into which people, in order to identify and link to learning resources, can put the terms about which they wish to learn more. The users can search for materials according with their information needs.

Resources identified were assessed by one person for relevance, and target audience and reviewed by a second person. Collected resources were assessed by members of the consortium for the following domains: correctness of the materials (correct and complete; correct but not complete; not quite correct; incorrect on an important matter); how much prior knowledge assumed (4-point scale from none to advanced knowledge required); language level; engagement level; educational value; general or specific topic covered.). Twenty-five health service users from the Cochrane Consumer Network also assessed the content online and gave free-text comments and a star rating. Multiple resources were allocated to them by the topic areas in which they had expressed an interest so that they could compare resources addressing similar issues. The data were collected using the Web 2.0 functionality of the database. A link to an instructional video, which also illustrates how the database works, was sent to all volunteers (http://bit.ly/15iEw7F, last accessed 8 September 2015).

### Development phase: materials

Starting from the review of the resources, the ECRAN materials – the website, the film, the educational games, for example – were developed through a process of peer review among partners, patients, and the public. The ECRAN film was developed in collaboration with RAI-SuperQuark, the longest and most successful scientific programme on Italian public television.

The accessibility and readability of the website were assessed during its early development (interim evaluation about structure and navigation using a three-language version, English, Italian, and Danish, by experts in health communication) and again just before its publication. The latter test was done by 20 users, selected by the European AIDS Treatment Group [17], the Cochrane Consumer Network [18], Oxford University Hospital, and IRCCS Istituto Mario Negri-PartecipaSalute [19].

We also ran a user test of the educational games in a secondary high school in Milan, where students and teachers were asked to consult and comment on the materials published on the ECRAN website.

### Dissemination phase

To ensure efficient dissemination, all the materials developed for lay people have been translated and dubbed into at least six European languages. All materials and tools are published under a Creative Commons licence on the ECRAN website (http://www.ecranproject.eu).

### Ethics approval

Due to the nature of the project and the activities no ethics committees approval was required.

## Results

### Exploratory phase: survey and database

A total of 1852 completed survey responses were received. Although the survey was directed at lay people, less than half the respondents were health service users, patients, parents or carers. The other responders were health advocates (e.g. health organisations), medical or allied health professionals, health educators, policy-makers or librarians. The overwhelming majority had a college/university-level qualification or higher. Nonetheless, over half of the respondents said their main reason for seeking health information was for personal use. Three quarters of respondents were women.

**Fig. 2:**
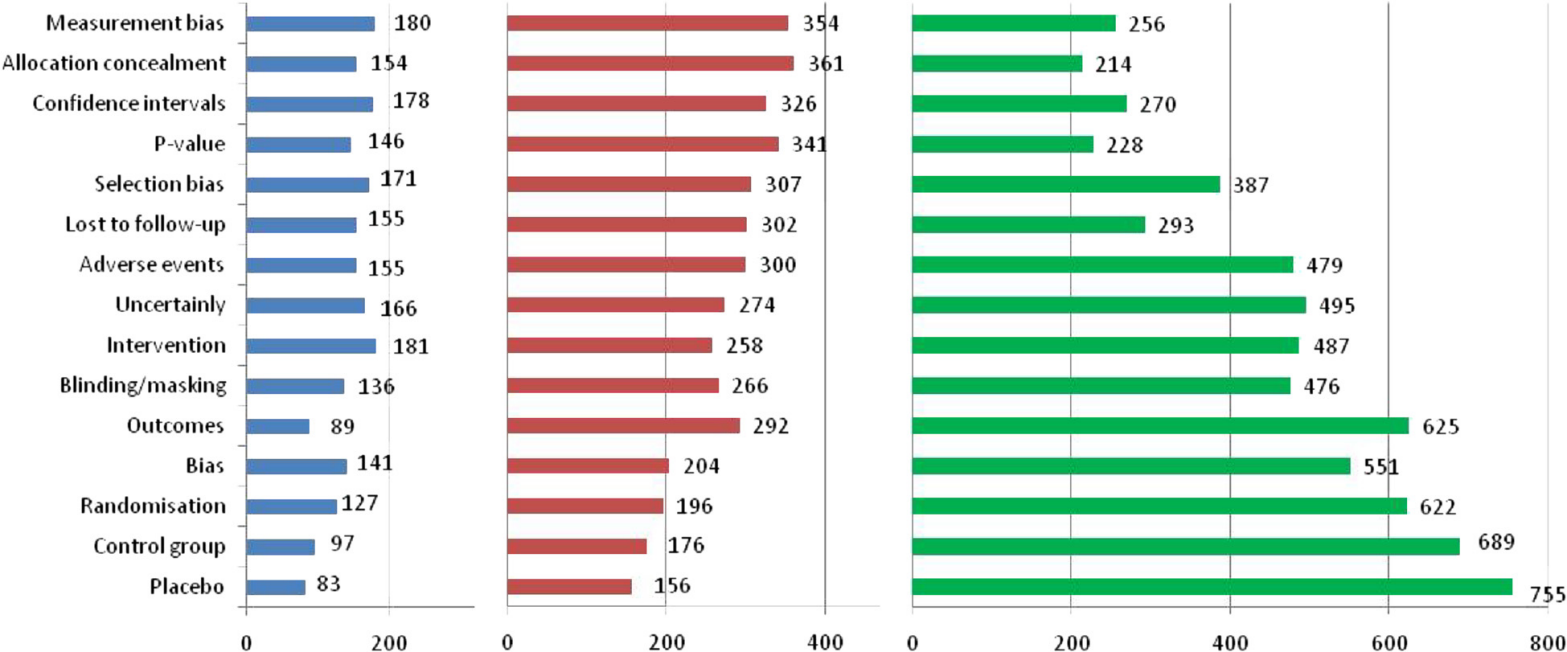
Requests for more information on the following terms. Blue: slightly interested in learning more. Red: very interested in knowing more. Green: I already understand this concept

The survey provided a glimpse into the lack of public understanding about how clinical trials are conducted. While most participants (73 % or 1358/1852) understood why clinical trials are necessary, over half (57 % or 1060/1852,) understood ‘little or not at all’ about the four pre-licensing phases. Figure 2 ranks the needs and preferences of the respondents with respect to key concepts involved in understanding clinical trials, according to the total number of positive responses.

Regarding the collected resources, most were judged suitable for beginners and less than 5 % required advanced knowledge. The majority used language suitable for a lay audience, about a third assumed some understanding of technical terms. Few resources were assessed as engaging and a third were classified as ‘serious and dull’. Most materials were generic but about a third of the resources used examples of specific conditions or were related to specific conditions. The ratings of responders were generally good and free-text comments ranged widely in content but were predominantly positive.

Ten learning resources were rejected as incorrect on an important matter. After that, 181 unique online learning resources through English-speaking patient groups, an additional 45 through the National Health Service Choices website, and 79 through ECRAN partner organisations were identified. The database includes open educational resources, websites, videos, books, interactive applications, or Wikipedia chapters. Our general conclusion was that further resources were needed and they needed to be more engaging.

### Development phase: materials

Several materials were developed by the ECRAN project, available at www.ecranproject.eu, including the Koha database. The materials differed according to target users as shown in Table 1. Although we received suggestions for improving translations of materials on our website during the development phase, we did not receive any negative comments about the style or our approach to the topics covered.

**Table 1:** European Communication on Research Awareness Needs (ECRAN) materials according to the ECRAN project’s targets

Materials	No. of languages	Main target^c^			
		General population	Clinicians and researchers	Journalists	Young people
Online database	23	✓	✓	✓	
Animated film^a^	25	✓	✓	✓	✓
Discover randomised clinical trials: interactive tutorial, FAQs, glossary, leaflet, etc.	6	✓	✓		
Serious games	6				✓
Testing Treatments interactive sibling websites^b^	9	✓			✓
Website	6	✓	✓	✓	✓
Facebook/Twitter	1	✓	✓	✓	✓
Media section	1			✓	

^a^23 official European languages, plus Norwegian and Luxembourgish versions

^b^English, Croatian, French, German, Italian, Norwegian, Polish, Portuguese, Spanish

^c^When the material is not addressed to a target, the related box is blank

*FAQs* frequently-asked questions

**Fig. 3:**
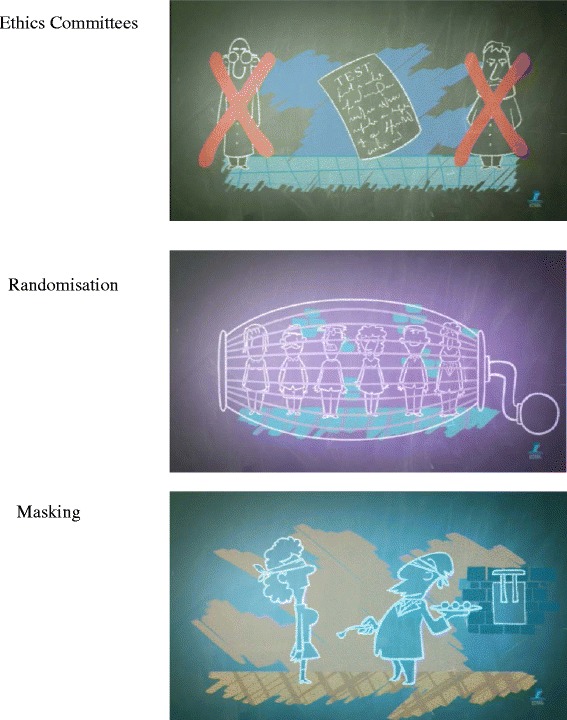
Examples of pictures used in the animated film, tutorial, and educational games. Legend: graphics and animation by Studio Bozzetto & Co.

The *animated film* [20] starts with the story of Scottish naval surgeon, James Lind, and his quest to learn how to treat scurvy by conducting an intervention experiment in 1747, hailed today as one of the earliest clinical trials in the world. The film’s modular structure allows either the whole film or its eight different modules to be displayed, covering: a clinical trial, ethics committees; randomisation; double blinding/masking; analysing the data; ‘one trial is not enough’; outcomes to be important to patients; and some pitfalls. Examples of images of the film are in Fig. 3.

*About clinical trials* [21] is a section of the website with different documents. These include a 76-page original interactive tutorial, in which the phases of clinical research are described in simple language, with a self-evaluating test.

In this section, several glossaries have been assembled using information collected in the database of resources and the analysis of websites. No ECRAN glossary was developed because many good-quality glossaries are already available through academic institutions, scientific societies, patients’ organisations, or other European projects. At the voice glossary of the ECRAN website a list of available glossaries with related links is available for each of the six languages of the project. The list includes ten glossaries in English, six in Italian, two in French, seven in German, two in Spanish, and one in Polish selected on the basis of the information collected in the database of resources, an analysis of existing websites, and suggestions from partners. The frequently-asked questions (FAQs) section includes an original document. It is one of the most challenging materials, since it endeavours to explain in detail, using plain language, the essential aspects of clinical trials. This section addresses common doubts and the questions raised by patients potentially interested in participating in clinical trials. The principal topics are: clinical trial phases; the importance of clinical research; risks and benefits of clinical trials; clinical trial participation (who can participate, who can be contacted); conscious participation and informed consent; and clinical trial funding.

Two *educational games* – ECRAN Lab and ECRAN Maze – were developed to attract the interest of young European people. The ECRAN Lab explains how a new drug is made: finding its ideal dose; checking its safety and effectiveness; comparing it to existing treatments; and looking at its adverse effects. The ECRAN Maze exploits the tablet accelerometer, since the movement of the tablet drives the path through the labyrinth. The game is organised in 12 levels, each corresponding to a question of increasing difficulty about clinical trials.

*Testing Treatments interactive sibling websites: Testing Treatments* is a book written for the public, covering many questions pertaining to clinical research [22, 23]. The book and translations (currently in a dozen other languages), together with audio materials, videos, and cartoons to enhance general knowledge of fair tests of treatments, are freely accessible from the Testing Treatments interactive [23] sibling websites [24]. The ECRAN website includes a direct link to each Testing Treatments interactive sibling site available in a European and other languages [25].

A *Media centre* [26] written by professionals, serves journalists and peer-to-peer dissemination of the ECRAN project across countries. The Media centre is exclusively in English. It has been conceived as a professional toolkit to introduce different aspects of clinical trials (methodological aspects; how to obtain reliable information; how to report results; how to explain technical terms using plain language).

The materials developed by the ECRAN project are available through its website which is accessible in multiple languages. Landing on the homepage, the net-surfer will find contents in the language automatically selected by his browser preferences.

### Dissemination phase

Some data on dissemination of the ECRAN project are reported in Table 2. As all the materials developed are available and downloadable by the ECRAN website it is difficult to know the number of people or organisations receiving the materials, as well as the geographic distribution. To celebrate the completion of the project and to facilitate dissemination about it, ECRIN and ECRAN organised a 3-day event in Luxembourg in 2014. Here the audience largely consisted of ECRIN and ECRAN participants. On 20 May 2014 the whole ECRAN project as well as the ECRAN video were presented to an international audience during the ECRIN and ECRAN celebrations of International Clinical Trials Day. The audience consisted of patients’ representatives, ECRAN partners, ECRIN researchers, international researchers, and journalists. On 21 May 2014 the different parts of the ECRAN project were presented in detail and discussed with the international audience, including representatives from patient organisations, researchers, and the press.

**Table 2:** Diffusion of European Communication on Research Awareness Needs (ECRAN) materials

Materials (period of analysis)	Results
Website access (March 2013 to August 2015)	Unique visitors: 35,151 average session duration: 1 m 50 s
Animated film (July 2013 to September 2015)	19,070 video views
	3270 YouTube views
	English, German, Italian and Spanish are the most used language
Serious games downloaded	361 ECRAN Lab
	344 ECRAN Maze
Open letter (petition) (May 2014 to August 2015)	439 signatures
Link to ECRAN web	1120 links from 85 sites
Congress, meeting, training course	35 (25 for scientific community, 13 for lay people)
Articles	
• in newsletter and lay press journals	23
• on website pages	38
Brochures distributed during congresses and events	1200 in English
	2000 in Italian
	200 in German
	200 in Spanish
	200 in French
	200 in Polish

Since the website was launched, two issues of a newsletter reporting ECRAN events have been published. The first issue (April 2014) focused on ECRAN materials and announced the meeting in Luxembourg; the second issue (August 2014) summarised the results of the project and tried to plan the future management and dissemination of the materials produced. The newsletters were sent to lists of journalists, journalism schools and European universities with relevant courses, patient organisations, and registered users.

## Discussion

Providing simple and independent information to patients and the public about clinical trials is important and challenging. Although some educational materials already exist, there has been no previous European initiative focussed specifically on promoting independent information for the public on clinical research. The need and demand for more information have been shown by the described survey despite the responders tending to have a higher educational level than the general public and the low response rate obtained – although a precise response rate cannot be calculated for open surveys on websites due to lack of denominator information.

Over the project duration of only 2 years, we have been able to organise a multidisciplinary, multinational consortium that produced and made freely available new materials to promote public knowledge about the importance of independent, international clinical trials in many languages.

The ECRAN project has developed materials to begin to meet this identified need, and these materials are now available for the general population. They are also available to clinicians and researchers to facilitate the discussion of clinical trials with patients. All the information materials reflect the principles of evidence-informed practice, and have been inspired by the objective of patient-centred medicine. The ECRAN materials have been developed within a public project using European Community funds and, all of them are freely available. The multi-lingual aspect of the ECRAN project needs to be stressed. Materials are available in six languages (English, French, Italian, Spanish, German, and Polish), which cover about 70 % of European citizens [27]. Some materials, such as the animated film, are available in the 23 official European languages. As the language of medical science is English, people who cannot understand English face a barrier to obtaining intelligible information. This has been partially overcome by ECRAN.

Dissemination of the ECRAN materials is clearly vital, but a widespread strategy directed to individuals and patients was incompatible with the aims and resources of this 2-year project. The impact of ECRAN materials should be assessed and we already have some encouraging information: the website, and the film in particular, have been covered in several articles for lay people, at medical congresses and workshops, and also linked from other websites, patients’ organisations included.

In the light of the successful research and development described in this article, it is important that the website is maintained and the inventory of resources is kept up to date as new materials are identified. This task can be delegated to stakeholders in the ECRAN consortium (clinicians, researchers, healthcare professionals, policy-makers, patient associations, communicators, journalists, etc.) and, in the future, to a new research project. Moreover, this task is integral to promoting efficiency in the programme of clinical trials being done by ECRIN, which was recently awarded the status of European Research Infrastructure Consortium (ERIC), to continue designing and conducting independent and multinational clinical trials throughout Europe. Continued updating of the inventory of relevant resources in the ECRAN website will make clear that researchers respect the information needs of past, present, and potential participants in their clinical trials.

ECRAN materials may also be used by ethics committees. These are responsible for ensuring that patients are given accurate and complete information to help them decide whether to participate in a clinical trial. However, it is often not easy to explain the trial or the technical terms involved. Ethics committees could consider referring patients to the ECRAN materials, and clinicians could also use them during medical visits when the nature and results of a clinical trial are explained or when patients are invited to participate.

Two educational games are among the materials developed by the ECRAN project to educate young European people about medical research and raise, more generally, their early awareness about scientific topics, a distinct novelty in the European context. Even with all limitations of this ‘first entry’ experience and the possibility of further improvement in both project and production, ECRAN Maze and ECRAN Lab may help to cover gaps in school education and programmes in several European countries. These gaps involve scientific subjects in particular, and more specifically knowledge of present-day topics (e.g. energy sources, urban environment and pollution, and medical and healthcare system topics which encompass the participation of the public and patients in clinical research).

The existence of these gaps was indirectly confirmed by another initiative of the ECRAN consortium, the open letter sent to the ministers of education [28]. The consortium expressed its concern about the scarce knowledge of clinical research and proposed steps to equip European people with better information and understanding. Since the younger generation will be crucial in addressing these gaps in knowledge, it was proposed that all high schools introduce, as part of their curricula, three 2-day education workshops over three consecutive years on the theme of clinical research. Materials from the ECRAN project, as well as from other ongoing initiatives, may be used to support these educational needs.

We expect that the information will reach both the general public and patient organisations because it is very important to involve patients and patients’ representatives in the design of clinical trials, and in using the information from clinical trials and systematic reviews in shared healthcare decision-making. The public needs access to information to help them understand clinical trials so that their participation will be meaningful and influential. We believe the ECRAN website makes an impressive beginning. The involvement of lay people and patients’ groups, such as the Cochrane Consumer Network and the European AIDS Treatment Group, both members of the ECRAN consortium, in dissemination is central to identify the best way to increase participation and the public debate in promoting better research for better healthcare.

During the development of the project, it became increasingly clear that the results obtained could contribute to the development of high-quality clinical research, in particular with joint initiatives with clinical groups, such as ECRIN. There was strong support from the participants at the ECRAN event to try to raise central funds to make ECRAN viable for the future given its multi-stakeholder representation and balanced view on evidence-based healthcare. Sustained funding for the ECRAN information platform could help to promote successful recruitment to independent clinical trials supported through ECRIN.

The continuing scandal of poor and wasteful medical research has emphasised the need for research funders and regulators, healthcare providers, clinical investigators, patients, and the public to work together to increase transparency and quality in research [13–15, 29–31]. In the near future, topics such as research priorities, important treatment outcomes, and trial design will be openly discussed among all parties involved to promote research of real value to patients and the wider community.

## Conclusions

Health literacy in both patients and public is needed to foster collaboration and participation. The ECRAN project has developed useful materials to facilitate understanding of the principles of clinical research, particularly independent clinical trials. However, this is not enough. Additional steps are needed to spread the culture of research and evidence-based healthcare, and so help to develop valid research for the common good. As the *BMJ* put it recently – ‘Let the patient revolution begin’! [32].

## Abbreviations

ECRAN: European Communication on Research Awareness Need
ECRIN: European Clinical Research Infrastructure Network
ERIC: European Research Infrastructure Consortium
FAQs: Frequently-asked questions
